# Comparative Characterization of Three Homologous Glutathione Transferases from the Weed *Lolium perenne*

**DOI:** 10.3390/foods13223584

**Published:** 2024-11-09

**Authors:** Annie Kontouri, Farid Shokry Ataya, Panagiotis Madesis, Nikolaos Labrou

**Affiliations:** 1 Laboratory of Enzyme Technology, Department of Biotechnology, School of Applied Biology and Biotechnology, Agricultural University of Athens, 75 Iera Odos Street, GR-11855 Athens, Greece; 2 Department of Biochemistry, College of Science, King Saud University, P.O. Box 2455, Riyadh 11451, Saudi Arabia; fataya@ksu.edu.sa; 3 Institute of Applied Biosciences, CERTH, 6th km Charilaou-Thermis Road, P.O. Box 361, Thermi, GR-57001 Thessaloniki, Greece; pmadesis@certh.gr

**Keywords:** abiotic stress, glutathione transferase, biotic stress, herbicide detoxification, *Lolium perenne*, *Lolium* sp.

## Abstract

The comparative analysis of homologous enzymes is a valuable approach for elucidating enzymes’ structure–function relationships. Glutathione transferases (GSTs, EC. 2.5.1.18) are crucial enzymes in maintaining the homeostatic stability of plant cells by performing various metabolic, regulatory, and detoxifying functions. They are promiscuous enzymes that catalyze a broad range of reactions that involve the nucleophilic attack of the activated thiolate of glutathione (GSH) to electrophilic compounds. In the present work, three highly homologous (96–98%) GSTs from ryegrass *Lolium perenne* (*Lp*GSTs) were identified by in silico homology searches and their full-length cDNAs were isolated, cloned, and expressed in *E. coli* cells. The recombinant enzymes were purified by affinity chromatography and their substrate specificity and kinetic parameters were determined. *Lp*GSTs belong to the tau class of the GST superfamily, and despite their high sequence homology, their substrate specificity displays remarkable differences. High catalytic activity was determined towards hydroxyperoxides and alkenals, suggesting a detoxification role towards oxidative stress metabolites. The prediction of the structure of the most active *Lp*GST by molecular modeling allowed the identification of a non-conserved residue (Phe215) with key structural and functional roles. Site-saturation mutagenesis at position 215 and the characterization of eight mutant enzymes revealed that this site plays pleiotropic roles, affecting the affinity of the enzyme for the substrates, catalytic constant, and structural stability. The results of the work have improved our understanding of the GST family in *L. perenne*, a significant threat to agriculture, sustainable food production, and safety worldwide.

## 1. Introduction

Weeds pose an important threat to agriculture worldwide. Metabolism-based herbicide resistance is a significant concern as it can provide resistance to current, future, and unknown herbicides [1,2,3]. Over the last twenty years, a significant rise in the number and variety of weeds that are resistant to herbicides has been observed [4,5,6]. This poses a threat to the long-term viability of agriculture on both local and worldwide levels. Metabolism-based herbicide resistance is linked to the increased expression of enzymes that detoxify herbicides, such as cytochrome P450 mixed-function oxidases (CYPs), family 1 UDP-glucose-dependent glycosyltransferases (UGTs), and GSTs, along with membrane-associated ATP-binding cassette (ABC) drug transporter proteins [1,2,3,7,8,9,10].

*L. perenne* is consistently included among the most serious weeds of winter cereal and perennial crops such as orchards, olive groves, vineyards, and alfalfa [4,5,6]. Weed competition in cultivated crops can reduce yield, making weed control a major concern in sustainable food production. *L. perenne* is especially troublesome because, if left uncontrolled, it grows quickly and competes aggressively for space, light, nutrients, and water. It can contaminate the seed harvest with undesirable seed, affecting both food quality and safety. The control of *L. perenne* relies mainly on herbicides that target the enzymes acetolactate synthase (ALS) and acetyl-CoA carboxylase (ACCase) [8]. These herbicides are linked with the highest risk of the rapid evolution of target-site resistance, which is most commonly due to mutations in the ALS or ACCase genes or to the expression of herbicide-detoxifying GSTs [2,7,8].

GSTs catalyze the addition of the sulfur atom of GSH (γ-L-Glu-L-Cys-Gly) to various endogenous and xenobiotic electrophile compounds [11,12,13]. The primary function of GSTs is their participation in the detoxification and the elimination of xenobiotic substrates including pesticides. In addition to transferase activity, GSTs also perform other biosynthetic roles [14] and noncatalytic activities such as ligand binding and transport [15,16,17,18].

Extensive studies have been conducted on the GSH-conjugating activities of GSTs towards various pesticides, contributing a significant role in determining herbicide selectivity in crops and weeds such as *A. myosuroides* and *L. rigidum* [19,20,21,22]. For example, a GST isoenzyme with high glutathione peroxidase activity has been proven to contribute to resistance to certain herbicides in resistant populations of *A. myosuroides* and *L. rigidum* [19,20,23,24,25,26,27]. This GST contributes in herbicide resistance by alleviating the oxidative stress through the breakdown of cytotoxic hydroperoxides that arise from herbicide-induced damage [23]. The enzyme from the black-grass *A. myosuroides* was found to have a limited capability to directly detoxify herbicides; however, it is involved in the metabolic regulation of protective flavonoids. The enzyme contributes indirectly to herbicide resistance by enhancing the accumulation of protective antioxidant flavonoids [23].

The plant GSTs are classified into different classes based on their sequences and structure relatedness [28,29]. The majority of plant GSTs are classified as tau (GSTU) and phi (GSTF) [13,28,29]. GSTs typically function as dimers consisting of two 24–29 kDa subunits; however, GSTs that belong to the lambda class or to the dehydroascorbate reductase class act as monomers. Experimentally determined crystal structures of all GSTs have demonstrated that the xenobiotic compounds bind at a structurally varied C-terminal hydro-phobic domain (named H-site) and a conserved N-terminal GSH binding domain (named G-site) [13,30,31]. Unlike the G-site, the H-site is less specific in terms of substrate types, allowing for the binding of substrates with diverse and different structures [13,30].

In the present work, we investigated the functional and catalytic features of three isoenzymes of the tau-class GST family from ryegrass *L. perenne*. *Lp*GSTs can contribute to herbicide resistance, either directly through the GSH-dependent detoxification process of herbicides or indirectly by playing a regulatory role in plant metabolism and antioxidant stress control. The study of *L. perenne* GSTs can provide valuable information on weed control and herbicide management strategies, impacting food security, sustainable food production, and the environment. To the best of our knowledge, this was the first study on the GST family of enzymes from *L. perenne*.

## 2. Materials and Methods

### 2.1. Molecular Cloning

Total mRNA from the leaves of *L. perenne* were isolated using the NucleoSpin RNA kit from MACHEREY-NAGEL GmbH & Co (Düren, Germany). The mRNA was reverse-transcribed (Thermo Fisher Scientific, Waltham, MA, USA) and the cDNA was used for the PCR reactions for the amplification of the coding sequence of the three GST isoenzymes (*Lp*GSTU25, *Lp*GSTU2, *Lp*GSTU5) using the following primers:

GSTAF1: 5′ ATG GCG TCC GAG AAA AGC AGC 3′;

GSTAR1: 5′ CT ACT CGA TGC CGT ACT TTT 3′;

GSTAF2: 5′ ATG GCG TCC GAG AAG AGC AGC 3′;

GSTAR2: 5′ CT ACT CGA TGC CGT ACT TCT 3′.

The PCRs (50 μL) for *Lp*GSTU25 and *Lp*GSTU5 clones were achieved using the AF1-AR1, AF2-AR2 (8 pmole each), template cDNA (0.5 μg), dNTPs (50 mM), 5 μL 10× buffer, and Taq DNA polymerase (1 unit). The PCR procedure comprised 30 cycles of 30 s at 94 °C, 30 s at 50 °C, and 1 min at 72 °C. The PCR for *Lp*GSTU2 was carried out in a total volume of 50 μL that contained the following: 8 pmole of each primer (AF2-AR2), 1 μg template cDNA, 50 mM dNTPs, 5 μL 10× buffer, and 1 unit of Taq DNA polymerase. The PCR procedure comprised 30 cycles of 30 s at 94 °C, 1 min at 44 °C, and 1 min at 72 °C. The final extension at 72 °C for 10 min was performed after the 30th cycle. The PCR products were ligated to the pCR^®^TOPO^®^ plasmid. PCR and the same primers were used to amplify the cloned ORFs. The PCR products were cloned into the pEXP5-CT/TOPO^®^TA plasmid, sequenced, and used to transform competent *E. coli* BL21(DE3) cells.

### 2.2. Bioinformatics and Structural Analysis of LpGSTU25

Structure prediction of *Lp*GSTU25 was achieved by AlphaFold [31]. Sequence alignments were carried out using the Clustal Omega program [32,33] and the sequences were analyzed using ESPript and ENDscript [34]. Phylogenetic tree was produced employing Geneious and iTOL 5 [35]. PDB files were inspected using UCSF Chimera 1.16 [36] and PyMOL [37].

### 2.3. Expression and Purification

Expressions of *Lp*GSTU25, *Lp*GSTU2, and *Lp*GSTU5 were achieved using *E. coli* BL21(DE3) cells at 37 °C in 1L LB medium containing ampicillin (100 μg/mL) according to [24]. Purification of the enzymes was accomplished according to [24].

### 2.4. Assay of Enzyme Activity, Kinetics Analysis, and Protein Determination

Enzyme assays and protein determination were performed according to published method [24]. Steady-state kinetic measurements were performed according to published methods [24,27] and analyzed using GraphPad Prism v5.

### 2.5. Site-Saturation Mutagenesis

Site-saturation mutagenesis was achieved according to [38] using, as templates, the cloned wild-type gene *Lp*GSTU25 and KAPA HiFi DNA polymerase (KapaBiosystems, Wilmington, MA, USA). The pairs of oligonucleotide primers used in the PCR reactions were as follows:

Mutant Forward primer: 5′- GTC TAC GAC NNN ATC GGC GTC C -3′; Mutant Reverse primer: 5′- GAC GCC GAT NNN GTC GTA GAC C -3′. A library of mutant enzymes was created and expressed in *E. coli* BL21(DE3) (20 mL LB medium containing 100 μg/mL ampicillin). Activity screening using, as substrates, CDNB/GSH allowed the selection of eight clones with the highest activity. Sequence analysis of the mutant clones revealed that the residue at position 215 was mutated to Ser, Val, His, Lys, Leu, Arg, Thr, and Tyr. The resulting mutant enzymes were expressed in *E.coli* BL21(DE3) and purified using affinity chromatography as described for the wild-type enzyme. The purified enzymes were subjected to steady-state kinetic analysis using the CDNB and GSH as substrates.

### 2.6. Thermal Stability

The operational stability of *Lp*GSTU25 and its mutants were determined in 0.1 M potassium phosphate buffer, pH 7, after heating the enzymes (20 to 85 °C) for 10 min. The T_m_ values (T_m_ is the temperature at which the enzyme loses 50% activity) were calculated from the graph of remaining activity (%) against temperature (°C) against relative inactivation (%). The data were analyzed by GraFit 3.0 and GraphPad Prism v5.

The time course of thermal inactivation of *Lp*GSTU25 and its mutants was studied in 0.05 M potassium phosphate buffer, pH 7. The rate of inactivation was measured by periodically removing samples for assay of enzymatic activity. Rate constants for a thermal inactivation were calculated from the graph of % remaining activity versus time (min) using the following equation:

$$\mathrm{Remaining}\;\mathrm{activity}=(1-F)e^{-k_{fast}t}+Fe^{-k_{slow}t}$$

Here, *F* represents the fractional residual activity of the partial active enzyme intermediate. *k_fast_* and *k_slow_*, are the rate constants for the slow and fast phase of the reaction. Analysis was achieved using GraphPad Prism v5.

### 2.7. Viscosity Dependence of Kinetic Parameters

The effect of viscosity on *k_cat_* was assayed at different glycerol concentrations (0–40% *v*/*v*) in 0.1 M potassium phosphate buffer, pH 6.5. Viscosity values were measured based on [39].

## 3. Results and Discussion

### 3.1. Cloning, Expression, and Substrate Specificity of LpGSTUs

**Figure 1:**
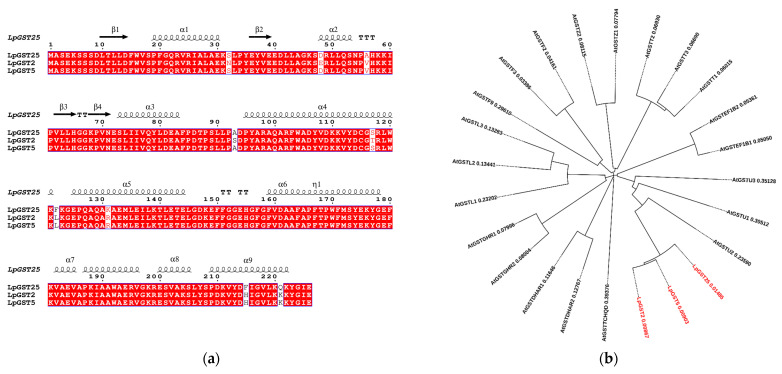
(**a**) Multiple-sequence alignments of *Lp*GSTU25, *Lp*GSTU2, and *Lp*GSTU5. Sequences were aligned with CLUSTAL Omega [33] and displayed using ESPript 3 [34]. *Lp*GSTU25 numbering is shown above the alignment. The predicted secondary structure of *Lp*GSTU25 is shown above the alignment. (**b**) Phylogenetic analysis of *Lp*GSTU25, *Lp*GSTU2, and *Lp*GSTU5 (labeled red) with sequences representing all the identified GST classes from *Arabidopsis thaliana*: phi (GSTF), tau (GSTU), lambda (GSTL), theta (GSTT), dehydroascorbate reductase (DHAR), elongation factor 1Bγ (EF1Bγ), zeta (GSTZ), Tetrachloro-hydroquinone dehalogenase (TCHQD), and Glutathionyl hydroquinone reductase (GHR). Sequences were aligned with CLUSTAL Omega [33] and phylogenetic tree was constructed with iTOL v5 [35]. The accession numbers of the proteins were as follows—phi class: AtGSTF1 (NP_180643.1), AtGSTF2 (NP_192161.1), and AtGSTF3 (NP_178394.1); tau class: AtGSTU1 (NP_176178.1), AtGSTU2 (NP_565178.1), and AtGSTU3 (NP_177598.1); lambda class: AtGSTL1 (NP_191064.1), AtGSTL2 (NP_001119157.1), and AtGSTL3 (NP_195899.1); theta class: AtGSTT1 (NP_198937.1), AtGSTT2 (NP_198940.3), and AtGSTT3 (NP_198938.1); DHAR class: AtGSTDHAR1 (NP_173387.1) and AtGSTDHAR2 (NP_177662.1); EF1Bgamma class: AtGSTEF1B1 (NP_563848.1) and AtGSTEF1B2 (NP_176084.1); zeta class: AtGSTZ1 (NP_178344.1) and AtGSTZ2 (NP_178343.1); TCHQD class: AtGSTTCHQD (NP_177853.1); GHR class: AtGSTGHR1 (NP_199315.1) and AtGSTGHR2 (NP_001031671.1).

In silico homology searches (BLASTp analysis) using the amino acid sequence of the GST from *Triticum aestivum* (accession number XP_044393881.1) as a query sequence revealed the presence of three homologous enzymes (96–98% homology) (Figure 1a). The full-length cDNAs with complete open reading frames of the three putative GSTs were isolated using RT-PCR. These isoenzymes (denoted *Lp*GSTU25, *Lp*GSTU2, and *Lp*GSTU5) display high amino acid sequence identity and therefore provide an excellent opportunity for studying structure–function relationships. A phylogenetic analysis was carried out to investigate the genetic connection between *Lp*GSTUs and GSTs from all known classes (Figure 1b). In plants, GSTs are divided into at least 14 classes [40], and our analysis revealed that the deduced amino acid sequences of the *Lp*GSTUs share a high degree of similarity with GSTs that belong to the tau class (Figure 1b). The tau-class GSTs are known to play a vital role in a wide range of catalytic and regulatory functions related to the detoxification of xenobiotics and the response to oxidative stress [13,41].

BLAST search using, as a query, either of the *Lp*GSTU sequences, revealed the presence of eight homologue GST sequences in Lolium species, with identities between 94.7 and 99.1%. Five sequences were from *L. perenne* (accession numbers: XP_051177894.1; AMY26593.1, XP_051177895.1; AMY26592.1; XP_051177900.1), one from *L. multiflorum* (accession number KAK1643535.1), and two from *L. rigidum* (accession numbers: XP_047090606.1; XP_047090605.1). The sequences with accession numbers AMY26592, AMY26593.1, and XP_051177894 corresponded to *Lp*GSTU25, *Lp*GSTU2, and *Lp*GSTU5, respectively. Multiple-sequence alignment of eight *Lp*GSTs is illustrated in Figure S1.

The coding sequences of *Lp*GSTUs were cloned into the pEXP5-CT/TOPO^®^TA plasmid to enable their expression in *E. coli* under the control of the T7 promotor. Following expression, single-step column affinity chromatography on GSH-Sepharose was used for the purification of the recombinant *Lp*GSTs (see Figure S2).

To reveal possible catalytic activities related to their biological roles, we examined the substrate specificity of the purified *Lp*GSTUs using a wide range of electrophilic substrates. The results (Table 1) showed that *Lp*GSTUs exhibit diverse substrate specificities and catalyze a broad spectrum of reactions. They showed appreciate catalytic activity with 17 out of the 20 diverse substrates tested. In general, *Lp*GSTUs display high activity towards halogenated compounds such as 1-chloro-2,4-dinitrobenzene (CDNB), 4-chloro-7-nitrobenzofurazan (NBD-chloride), and p-nitrobenzyl-chloride (pNBD). The enzymes did not show any activity using the herbicide fluorodifen as a substrate, in contrast to other tau-class GSTs [42]. The GSH-dependent hydroperoxidase function of *Lp*GSTUs was also assessed using cumene hydroperoxide or tert-butyl hydroperoxide as substrates. Cumene hydroperoxide appeared to be the best substrate between the two peroxides examined. The notable hydroperoxidase function of *Lp*GSTUs may have been related to their role in conferring tolerance to oxidative-stress toxic metabolites [43,44]. Oxidative stress also leads to the formation of cytotoxic alkenals such as trans-2-nonenal. Alkenals react with GSH through a Michael addition to the α,β-unsaturated carbonyl group to form conjugates. [45]. *Lp*GSTUs effectively detoxify and eliminate trans-2-nonenal, trans-4-phenyl-3-buten-2-one, and ethacrynic acid (2-[2,3-Dichloro-4-(2-methylidenebutanoyl)phenoxy]acetic acid).

GSTs also showed antioxidant function through their dehydroascorbate reductase and thioltransferase activities [46]. *Lp*GSTUs efficiently catalyze the reduction of dehydroascorbate to ascorbic acid. They also displayed thioltransferase activity using 2-hydroxyethyl disulphide (2,2-dithiodiethanol) as a substrate. It is widely accepted that during oxidative stress, protein thiols can undergo S-thiolation, leading to the creation of protein-thiol disulphides. These reactions serve regulatory and/or protective roles [47].

Isothiocyanates are organic compounds that are produced by plants in response to various forms of stress or injury [48]. These compounds are formed through the breakdown of glucosinolates by an enzyme called thioglucosidase, also known as myrosinase [48,49]. Recent studies have shown that GSTs play a crucial role in the metabolism of isothiocyanates by catalyzing the conjugation of naturally occurring isothiocyanates with GSH, resulting in the formation of dithiocarbamates [49,50]. *Lp*GSTUs are able to catalyze the reaction between GSH and the two model isothiocyanates, the phenethyl isothiocyanate and the allyl isothiocyanate.

### 3.2. Kinetic Analysis

Steady-state kinetics analysis of *Lp*GSTUs was conducted using, as a substrate, the model halogenated aromatic compound CDNB. The k_cat_ and K_m_ parameters were measured and the results are listed in Table 2. The study revealed that when the concentration of GSH was varied, *Lp*GSTU25, *Lp*GSTU2, and *Lp*GSTU5 obeyed Michaelis–Menten kinetics (Figure S3a–c). The K_m_ values for GSH fall within the range observed for other tau-class GSTs [13,24,25,27]. When the concentration of CDNB was varied (Figure S3d–f), all but *Lp*GSTU5, obeyed Michaelis–Menten kinetics. The steady-state kinetic analysis of *Lp*GSTU5 revealed a sigmoid dependence on CDNB concentration (Figure S3f). The initial velocity data were well fitted to a rate equation for positive cooperativity between the two H-sites and Hill coefficient (n_H_ value) of 1.94 ± 0.1. It is well known that the kinetic behavior of several GSTs that belong to the tau and phi classes deviates from the normal Michaelis–Menten kinetics, obeying allosteric kinetics [27]. Previous investigations, based on x-ray crystallography, have established that key residues that bridge the dimer interface can form a network of interactions, allowing the intersubunit communication of H-sites [42]. The exact biological function of the positive cooperativity observed in several tau- and phi-class GSTs is still not fully understood [13,27,42]. However, it is thought that GSTs display significant catalytic power in metabolizing and eliminating potential toxic compounds that the cell may encounter. It is believed that cooperativity offers a detoxification benefit in situations where the cell is at risk from harmful substances [42].

### 3.3. The Effect of Viscosity on K_cat_

**Figure 2:**
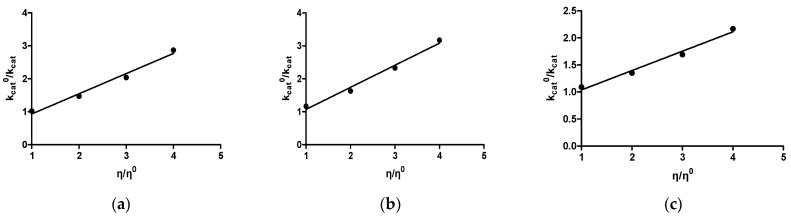
The impact of viscosity on k_cat_ for the CDNB/GSH reaction catalyzed by *Lp*GSTU25 and the mutant enzymes Phe215Ser and Phe215Lys. Plots of k^0^_cat_/k_cat_ as a function of η/η^0^ with glycerol as cosolvent for the wild-type (**a**), and for the Phe215Ser (**b**) and Phe215Lys (**c**) mutant enzymes, are shown.

The effect of viscosity on k_cat_ was studied to shine light on the rate-limited step of the catalytic reaction. Previous investigations have shown that in the majority of GSTs, the rate-limited step is relevant to product release or to the diffusion-controlled structural transitions of the protein [51,52,53]. When the product release is restricted by a strictly diffusional barrier, the inverse relative rate constant, k^0^_cat_/k_cat_ (k^0^_cat_ is determined at viscosity η^0^), when plotted against the relative viscosity, η/η^0^, is linear with a slope approaching unity. However, if the product release is restricted by chemistry or another non-diffusional barrier, the slope will be nearly zero [51,52,53]. As shown in Figure 2a for the wild-type enzyme, when the medium viscosity is increased, the k_cat_ is decreasing with a slope of 0.61 ± 0.060, suggesting that diffusion-controlled structural rearrangements of the protein determine the rate-limiting step.

### 3.4. The Role of Phe215 in Xenobiotic Substrate Binding and Catalysis

**Figure 3:**
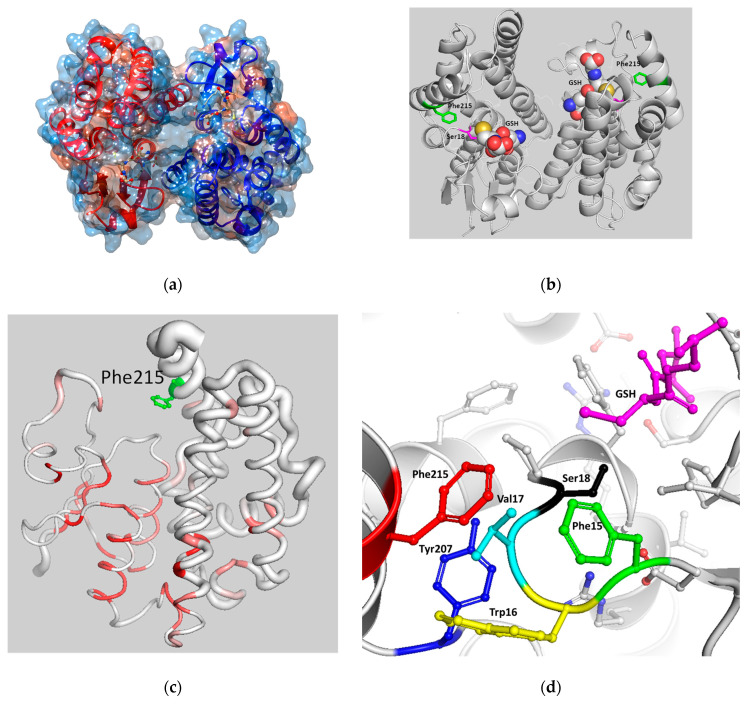
(**a**) Surface representation of *Lp*GSTU25. Each subunit is displayed in a distinct color (red–blue). The ball-and-stick representation displays the bound GSH, colored based on the atom type. The figure was created using the program UCSF Chimera 1.16. [36]. (**b**) Ribbon representation of *Lp*GSTU25 dimer. The spheres representing the bound GSH have been colored based on the atom type. Phe215 is depicted and colored green. The figure was created by PyMOL [37]. (**c**) *Lp*GSTU25 model in PyMOL ‘Sausage’ representation, with tube rendering where the radius represents the average RMS deviation per residue between Cα pairs. The tube is tinted based on the degree of sequence preservation, ranging from white (lower score) to red (matching identity). Phe215 is shown in a stick representation and has been colored green. The analysis was created by ENDscript [34] and the figure was created by PyMOL [37]. (**d**) Network of interactions between Phe215 (red), Trp16 (yellow), Val17 (turquoise), Ser18 (black), and Tyr207 (Blue). The bound GSH is displayed in a stick representation with a magenta color.

Among the three isoenzymes, *Lp*GSTU25 displays the highest catalytic activity towards the synthetic halogenated aromatic compounds (e.g., CDNB) compared to the other two isoenzymes. Therefore, *Lp*GSTU25 was selected for further structure–function studies. The prediction of the 3D structure of *Lp*GSTU25 was achieved using the AlphaFold [31] algorithm (Figure 3). The substrates binding sites (G-site and H-site) of *Lp*GSTU25 form a large open cleft (Figure 3a,b) and exhibit a high degree of sequence identity with *Lp*GSTU2 and *Lp*GSTU5 (Figure 1a and Figure 3c). Structural analysis of *Lp*GSTU25 revealed that the non-conserved residue Phe215 (replaced by His in *Lp*GSTU2 and *Lp*GSTU5, Figure 1a) is positioned towards the ligand binding site (Figure 3b,c), suggesting its potential role in substrate binding and/or catalysis. This amino acid residue is situated at the end of the C-terminal α-helix H9 (Figure 1a). This helix has been extensively studied in other GSTs and its structural and functional significance is well established [13]. Phe215 is involved in an extensive interaction network with the H-site residue Tyr207 and other residues that stabilize the N-terminal region (Figure 3d). The N-terminal region accommodates important residues (Trp16, Phe15, Val17) that play roles on G- and H-site formation, including the catalytic residue Ser18 (Figure 3d). The other non-conserved residue at the C-terminal α-helix H9 is Gln221, which has been substituted for Lys in *Lp*GSTU2 and *Lp*GSTU5. Gln221 lies outside the H-site and faces towards the solvent, indicating a limited functional/catalytic role.

Site-saturation mutagenesis was employed to investigate the role of Phe215. A library of mutant enzymes was created and expressed in *E. coli* BL21(DE3). An activity screening of the Phe215 mutant library was conducted, allowing the selection of eight clones with the highest activity. Sequence analysis of the mutant clones revealed that the residue at position 215 was mutated to Ser, Val, His, Lys, Leu, Arg, Thr, and Tyr. The resulting mutant enzymes were expressed in *E.coli* BL21(DE3), purified by affinity chromatography, and subjected to steady-state kinetic analysis using the substrates CDNB and GSH. The obtained data (Table 3) revealed moderate alterations in the K_m_ values of the mutant enzymes towards CDNB and GSH. On the other hand, the effect of mutations on k_cat_ values appeared to be substantial (6–10-times reduction) when compared to the wild-type enzyme (Table 3). The large effect of the mutations on the k_cat_ indicated the contribution of Phe215 on the rate-limiting step. Examining the effect of viscosity on the activity of two mutants, Phe215Ser and Phe215Lys, revealed a linear relationship with slopes 0.67 ± 0.06 and 0.36 ± 0.04, respectively (Figure 2b,c). The slopes obtained for the mutant enzymes were considerably altered compared to that of the wild-type enzyme (0.61 ± 0.06). This observation suggests that the mutations at the 215 position affects the rate-limiting step of the catalytic reaction. Previous investigations had established that two regions of particular importance that affect the rate-limiting step in GSTs are α-helix 2 and the C-terminal α-helix 9 [51,52,53].

### 3.5. Effect of Mutations on Structural Stability

**Figure 4:**
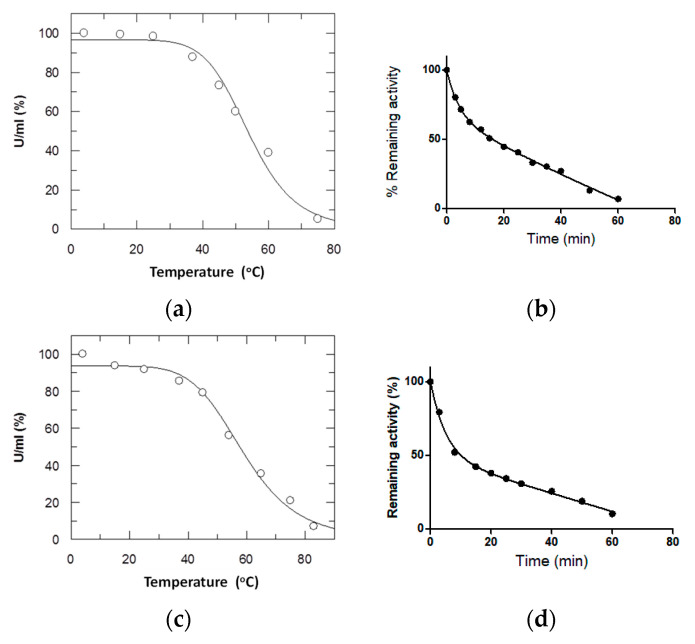
Thermal inactivation of *Lp*GSTU25 (**a**) and Phe215His mutant enzyme (**c**). The residual activities were measured after heat treatment of enzymes at different temperatures (°C) for 10 min. The course of thermal inactivation of *Lp*GSTU25 (**b**) and Phe215His mutant enzyme (**d**) determined at the T_m_ of each enzyme (see Table 4) are shown.

The melting temperature (T_m_) of the enzymes was determined through thermal denaturation experiments. This analysis allowed the rapid assessment of the mutations on protein stability. The T_m_ values obtained are summarized in Table 4. The results indicated that four point mutations (Phe215His, Phe215Ser, Phe215Arg, and Phe215Lys) had a significant stabilizing effect on the enzyme. Conversely, the mutations Phe215Val and Phe215Leu led to a considerable decrease in protein stability compared to the wild-type enzyme. On the other hand, the mutations Phe215Thr and Phe215Tyr had a minimal effect on the enzyme’s stability. Interestingly, the mutant Phe215His showed a substantial increase in thermal stability (Figure 4a,b). To confirm this finding, the analysis of the kinetics of thermal inactivation of the wild-type along with that of Phe215His was carried out at a temperature equivalent to the T_m_ of each enzyme (55 °C and 58 °C, respectively; see Table 4). As shown in Figure 4b, the inactivation reaction for the wild-type enzyme proceeded in two distinct phases: an initial fast phase of inactivation and a slow phase. A biphasic model was the best fit for the inactivation kinetics. As illustrated with the wild-type enzyme, the relationship between the remaining activities and heating time can be accurately described by the combination of two exponential terms. Several enzymes display biphasic inactivation kinetics, which is markedly different from first-order kinetics. The inactivation rate constants (min^−1^) were calculated for the fast and slow phase of inactivation and the results along with the half-life values are listed in Table 5. The inactivation reaction for the Phe215His mutant enzyme showed a one-phase decay, indicating that the thermal inactivation mechanism of the mutant enzyme had been significantly affected by the mutation. Notably, the mutant enzymes with positive charged residues (Arg, Lys, His) at position 215 displayed enhanced T_m_ values and therefore thermostability. All these results suggest that position 215 is a significant structural determinant of thermal stability.

## 4. Conclusions

GSTs play a significant role in determining herbicide selectivity in crops and weeds such as *L. perenne*. The characterization of herbicide-metabolizing enzymes contributes significantly in weed management and control. In the present work, three homologue GSTs from *L. perenne* that belong to the tau class were characterized. *Lp*GSTUs display a high amino acid sequence identity (96–98% homology), providing an excellent opportunity for studying structure–function relationships. *Lp*GSTUs show broad substrate specificity and high activity against xenobiotics and oxidative stress by-products, suggesting a prooxidant protective function that is likely related to the minimization of oxidative damage and cell detoxification [54,55]. Structural studies complemented with site-saturation mutagenesis revealed that the amino acid residue at position 215 is a key structural determinant that affects substrate affinity, catalysis, and thermostability. The results of the present work shed light on the catalytic and functional role of the three members of the GST family from *L. perenne*, a key agricultural grass weed. The comparative analysis of the catalytic and functional properties of *Lp*GSTUs provided new crucial information on the enzymes’ structure–function relationships and evolution. The results of the work have improved our understanding of the GST family in *L. perenne*, key enzymes that can affect sustainable food production and safety.

## Figures and Tables

**Table 1:** Specific activity of purified *Lp*GSTUs. The data represent the means of triplicate determinations with variation less than 5% in all cases.

Substrate	Special Activity (U/mg)		
	*Lp*GSTU-25.1	*Lp*GSTU-5	*Lp*GSTU-2
CDNB (1-chloro-2.4 dinitrobenzene)	66.3	2.0	2.7
NBD-chloride (4-Chloro-7-nitrobenzofurazan)	3.9	2.5	0.6
pNBD (p-Nitrobenzyl-Chloride)	9.8	9.5	2.1
CuOOH(Cumene hydroperoxide)	1.5	4.1	0.9
Tert-butyl-hydroperoxide	0.5	0.8	0.4
HNE (Trans-2-nonenal)	7.2	20.9	0.2
2-[2,3-Dichloro-4-(2-methylidenebutanoyl)phenoxy]acetic acid (Ethacrynic acid)	10.3	36.2	2.6
Trans-4-phenyl-3-buter-2-one	0.4	0.5	0.3
2.2-Dithiodiethanol	3.3	6.5	2.2
DHA (Dehydroascorbate)	20.5	160.2	0.8
AITC (Allyl isothiocyanate)	5.2	25.8	6.9
PEITC (Phenethyl isothiocyanate)	19.5	48.0	1.3
Fluorodifen	0.0	0.0	0.1
Bromosulphopthalein	24.5	90.9	0.0
Sulphanilamide	0.0	0.0	0.1

**Table 2:** Steady-state kinetic analysis of *Lp*GSTUs for the CDNB/GSH substrate system.

Enzyme	k_cat_ (min^−1^)	K_m_ (mM) (GSH)	K_m_ (mM) (CDNB)	k_cat_/K_m_, (mM^−1^ min^−1^) (GSH)	k_cat_/K_m_, (mM^−1^ min^1^) (CDNB)
*Lp*GSTU25	6955 ± 140.0	1.14 ± 0.12	0.28 ± 0.03	6100 ± 869.40	24,839 ± 3704
*Lp*GSTU2	63.7 ± 1.2	0.46± 0.05	0.47 ± 0.03	138.5 ± 11.80	135.5 ± 13.60
**Enzyme**	**k_cat_ (min^−1^)**	**K_m_ (mM)** **(GSH)**	**S_0.5_ (mM) ^1^** **(CDNB)**	**k_cat_/K_m_ (mM^−1^ min^−1^)** **(GSH)**	**k_cat_/S_0.5_ (mM^−1^ min^1^)** **(CDNB)**
*Lp*GSTU5	78.4 ± 9.9	0.57 ± 0.03	0.10 ± 0.04	137.5 ± 10.20	784 ± 67.30

^1^ The Hill coefficient determined nH = 1.94 ± 0.10.

**Table 3:** Steady-state kinetic parameters of the wild-type *Lp*GSTU25 and its mutants for the CDNB conjugation reaction.

	k_cat_ (min^−1^)	Κ_m_ (mM) (GSH)	K_m_ (mM) (CDNB)	k_cat_/K_m_ (mM^−1^ min^−1^) (GSH)	k_cat_/K_m_ (mM^−1^ min^−1^) (CDNB)
*Lp*GSTU25	6955 ± 140.0	1.14 ± 0.12	0.28 ± 0.03	6100 ± 869.4	24,839 ± 3704.0
Phe215Thr	1650 ± 27.4	1.43 ± 0.16	0.31 ± 0.03	1153 ± 10.0	5322 ± 565.0
Phe215Val	460 ± 7.5	1.39 ± 0.13	0.29 ± 0.02	331 ± 3.1	1586 ± 150.0
Phe215Ser	364 ± 6.7	1.68 ± 0.14	0.33 ± 0.03	1103 ± 12.0	1103 ± 133.0
Phe215Leu	3.26 ± 1.1	1.35 ± 0.12	0.25 ± 0.02	2.4 ± 0.2	13.1 ± 1.2
Phe215Arg	468 ± 6.7	1.06 ± 0.17	0.92 ± 0.32	442 ± 0.3	509 ± 189.0
Phe215Tyr	2760 ± 114.0	1.03 ± 0.04	0.91 ± 0.12	2679 ± 182.0	3032 ± 585.6
Phe215Lys	1000 ± 34.4	1.98 ± 0.30	0.77 ± 0.07	505 ± 34.0	1298 ± 179.9
Phe215His	358 ± 11.8	1.41 ± 0.23	0.68 ± 0.08	254 ± 19.0	527 ± 89.3

**Table 4:** Melting temperatures of wild-type *Lp*GSTU25 and its mutant enzymes as determined by thermal denaturation experiments.

Enzyme	T_m_ (°C)
*Lp*GSTU25	54.1 ± 1.5
Phe215Val	50.3 ± 1.7
Phe215His	58.9 ± 1.6
Phe215Thr	54.9 ± 0.9
Phe215Ser	57.5 ± 0.9
Phe215Leu	51.8 ± 0.6
Phe215Lys	57.0 ± 0.9
Phe215Arg	56.3 ± 0.8
Phe215Tyr	54.8 ± 0.4

**Table 5:** Inactivation rate constants and half-life values for the melting temperatures of wild-type *Lp*GSTU25 and Phe215His mutant enzyme.

Enzyme	Inactivation Rate Constants (min^−1^)		Half-Life (min)	
	Fast Phase	Slow Phase	Fast Phase	Slow Phase
***Lp*GSTU25**	0.231	0.004	3.0	171.5
**Phe215His**	0.075	−	9.3	−

## Data Availability

The original contributions presented in the study are included in the article/Supplementary Materials; further inquiries can be directed to the corresponding author.

## Supplementary Materials

The following supporting information can be downloaded at: https://www.mdpi.com/article/10.3390/foods13223584/s1, Figure S1: Multiple-sequence alignments of eight GST sequences from *L.* species, identified by BLAST search using as a query either of the *Lp*GSTU sequences; Figure S2: SDS-PAGE analysis of purified *Lp*GSTU25, *Lp*GSTU2, and *Lp*GSTU5; Figure S3: Kinetic analysis of *Lp*GSTU25, *Lp*GSTU2, and *Lp*GSTU5 using GSH and CDNB as substrates.
