# Supplementary material for: Comparative Characterization of Three Homologous Glutathione Transferases from the Weed Lolium perenne
Source: Foods. 2024 Nov 9;13(22):3584. doi: 10.3390/foods13223584 (PMC11593036; doi:10.3390/foods13223584)
Supplement: Supplementary file 1 [file foods-13-03584-s001.zip › foods-3268420-Figure S3.pdf]

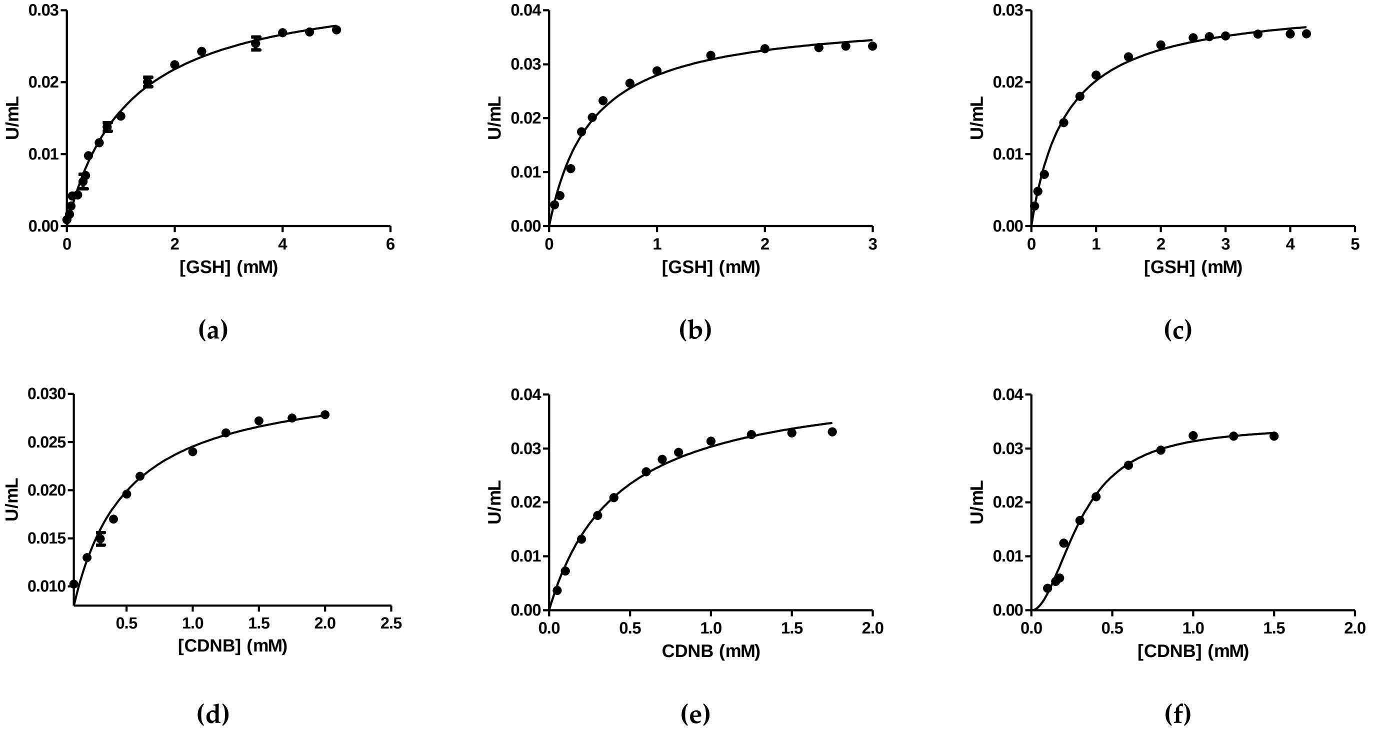

Figure S3. Kinetic analysis of *Lp*GSTU25 (**a**), *Lp*GSTU2 (**b**), *Lp*GSTU5 (**c**), using the GSH as a variable substrate and CDNB at a fixed concentration. Experiments were performed in triplicate. Kinetic analysis of *Lp*GSTU25 (**d**), *Lp*GSTU2 (**e**), *Lp*GSTU5 (**f**), using CDNB as a variable substrate and GSH at a fixed concentration. Experiments were performed in triplicate.
