# Supplementary material for: Comparative Characterization of Three Homologous Glutathione Transferases from the Weed Lolium perenne
Source: Foods. 2024 Nov 9;13(22):3584. doi: 10.3390/foods13223584 (PMC11593036; doi:10.3390/foods13223584)
Supplement: Supplementary file 1 [file foods-13-03584-s001.zip › foods-3268420-supplementary.pdf]

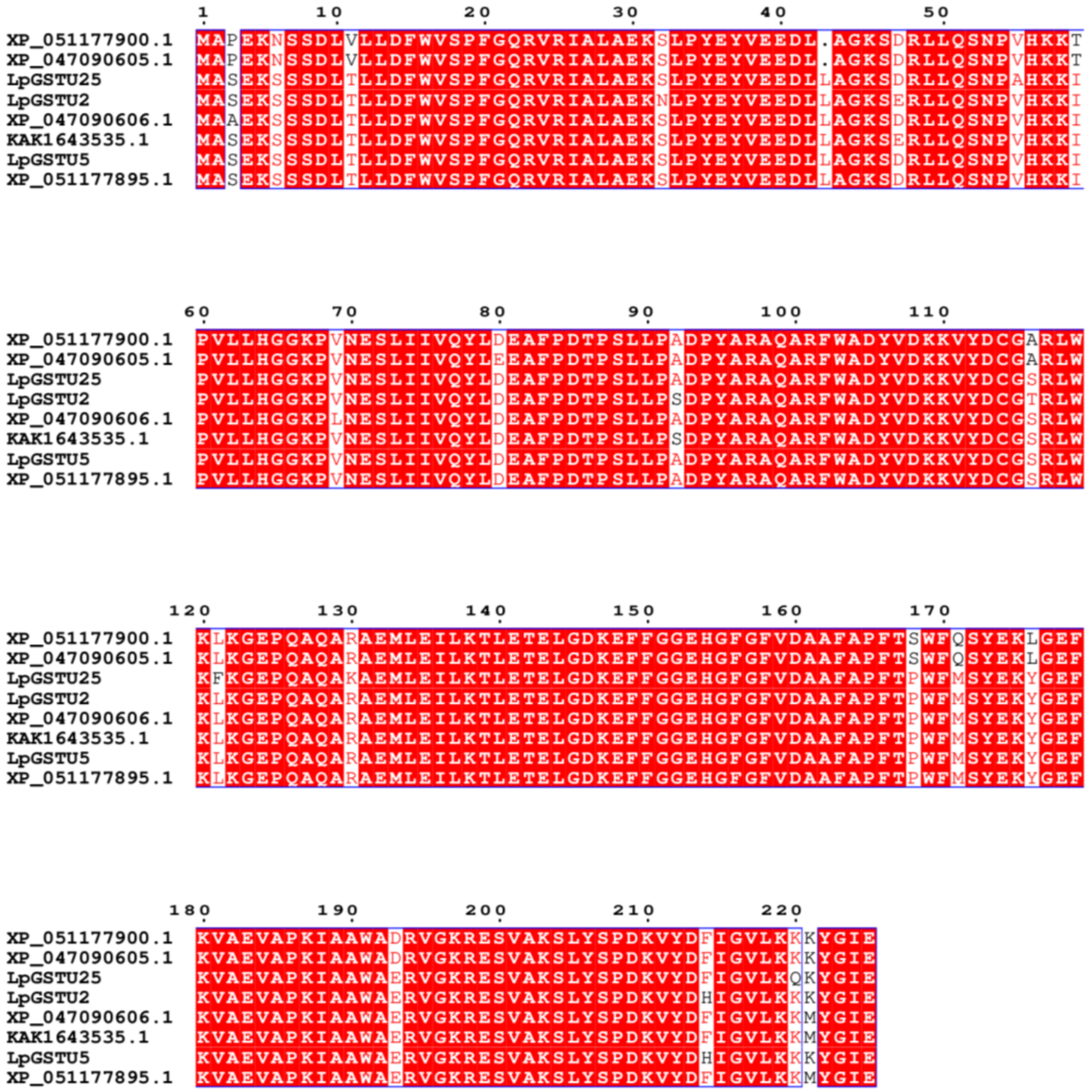

Figure S1. Multiple sequence alignments of eight GST sequences from Lolium species, identified by BLAST search using as a query either of the *Lp*GSTU sequences. Five sequences are from *Lolium perenne* (accession numbers: XP_051177894.1; AMY26593.1, XP_051177895.1; AMY26592.1; XP_051177900.1), one from *Lolium multiflorum* (accession number KAK1643535.1), and two from *Lolium rigidum* (accession numbers: XP_047090606.1; XP_047090605.1). The sequences with accession numbers AMY26592, AMY26593.1, XP_051177894 corresponds to *Lp*GSTU25, *Lp*GSTU2, *Lp*GSTU5 of the present study, respectively. Sequences were aligned with CLUSTAL Omega [33] and displayed using ESPript 3 [34]. Conserved areas are shown shaded. A column is framed, if more than 70% of its residues are similar according to physico-chemical properties.

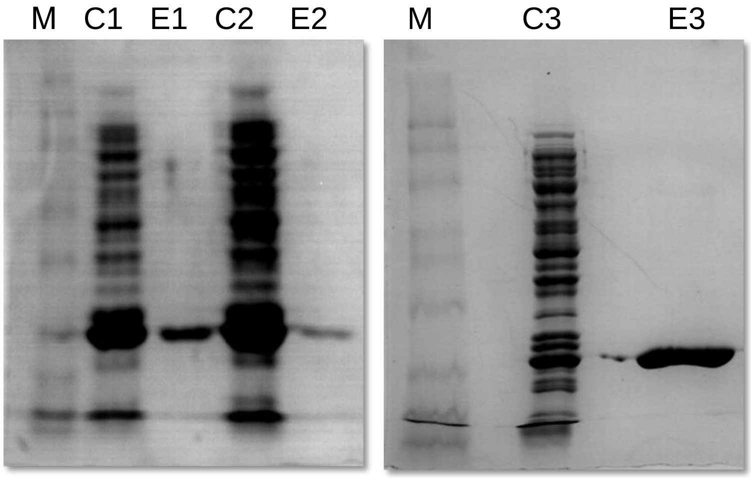

Figure S2. SDS-PAGE analysis of purified *Lp*GSTU25, *Lp*GSTU2, *Lp*GSTU5. Elution was carried out using GSH (10 mM). M protein markers. Lane C1, C2, C3: recombinant *E. coli* BL21(DE3) crude extracts, expressing *Lp*GSTU25, *Lp*GSTU2, *Lp*GSTU5, respectively. Lanes E1-E3 purified *Lp*GSTU25, *Lp*GSTU2, *Lp*GSTU5, respectively.
